# Diurnally Fluctuating *p*CO_2_ Modifies the Physiological Responses of Coral Recruits Under Ocean Acidification

**DOI:** 10.3389/fphys.2018.01952

**Published:** 2019-01-11

**Authors:** Lei Jiang, Ya-Juan Guo, Fang Zhang, Yu-Yang Zhang, Laurence John McCook, Xiang-Cheng Yuan, Xin-Ming Lei, Guo-Wei Zhou, Ming-Lan Guo, Lin Cai, Jian-Sheng Lian, Pei-Yuan Qian, Hui Huang

**Affiliations:** ^1^Key Laboratory of Tropical Marine Bio-resources and Ecology, South China Sea Institute of Oceanology, Chinese Academy of Sciences, Guangzhou, China; ^2^Guangdong Provincial Key Laboratory of Applied Marine Biology, South China Sea Institute of Oceanology, Chinese Academy of Sciences, Guangzhou, China; ^3^Hainan Tropical Marine Biology Research Station, Chinese Academy of Sciences, Sanya, China; ^4^University of Chinese Academy of Sciences, Beijing, China; ^5^ARC Centre of Excellence for Coral Reef Studies, James Cook University, Townsville, QLD, Australia; ^6^Shenzhen Research Institute and Department of Ocean Science, Hong Kong University of Science and Technology, Hong Kong, China

**Keywords:** ocean acidification, diurnal *p*CO_2_ fluctuations, coral calcification, carbonic anhydrase, proton pump, trade-off

## Abstract

Diurnal *p*CO_2_ fluctuations have the potential to modulate the biological impact of ocean acidification (OA) on reef calcifiers, yet little is known about the physiological and biochemical responses of scleractinian corals to fluctuating carbonate chemistry under OA. Here, we exposed newly settled *Pocillopora damicornis* for 7 days to ambient *p*CO_2_, steady and elevated *p*CO_2_ (stable OA) and diurnally fluctuating *p*CO_2_ under future OA scenario (fluctuating OA). We measured the photo-physiology, growth (lateral growth, budding and calcification), oxidative stress and activities of carbonic anhydrase (CA), Ca-ATPase and Mg-ATPase. Results showed that while OA enhanced the photochemical performance of *in hospite* symbionts, it also increased catalase activity and lipid peroxidation. Furthermore, both OA treatments altered the activities of host and symbiont CA, suggesting functional changes in the uptake of dissolved inorganic carbon (DIC) for photosynthesis and calcification. Most importantly, only the fluctuating OA treatment resulted in a slight drop in calcification with concurrent up-regulation of Ca-ATPase and Mg-ATPase, implying increased energy expenditure on calcification. Consequently, asexual budding rates decreased by 50% under fluctuating OA. These results suggest that diel *p*CO_2_ oscillations could modify the physiological responses and potentially alter the energy budget of coral recruits under future OA, and that fluctuating OA is more energetically expensive for the maintenance of coral recruits than stable OA.

## Introduction

Since the Industrial Revolution, about one third of human-emitted CO_2_ has been absorbed by the ocean, resulting in ocean acidification (OA), a phenomenon characterized by declines in seawater pH, carbonate concentration and saturation state of calcium carbonate (CaCO_3_) (Sabine et al., 2004). Relative to pre-industrial levels, average surface ocean pH has decreased by 0.1 units, and a further reduction of 0.3–0.4 units is projected by the end of this century based on “Business as usual” scenario (Orr et al., 2005; Gattuso et al., 2015).

Ocean acidification constitutes one of the most serious threats to various marine calcifying taxa because it reduces the availability of carbonate ions that are needed to accrete CaCO_3_ (Orr et al., 2005); among them reef corals, which construct and maintain complex reef framework structures, are extensively studied (Chan and Connolly, 2013; Kroeker et al., 2013). Numerous laboratory experiments have demonstrated negative yet variable effects of OA on coral skeletal growth, with a mean decline in calcification of 15% per unit decrease in aragonite saturation state (Ω_Arg_) (Chan and Connolly, 2013). In these empirical studies, reef corals have been exposed to static pH levels consistent with open ocean projections of seawater pH declines of 0.3–0.4 units by the year 2100. However, compared with pelagic ocean environments, the carbonate chemistry on coral reef ecosystems is highly dynamic. Large daily swings in seawater pH and *p*CO_2_, mainly driven by biological activities (photosynthesis and respiration), have been recorded in many reef locations around the world. During daytime, uptake of CO_2_ and ${\mathrm{HCO}}_{3}^{-}$ by photosynthesis decreases seawater *p*CO_2_ and elevates pH, while nighttime respiration decreases pH and increases *p*CO_2_. Natural variability in carbonate chemistry is particularly characteristic of the shallow coastal reefs (Rivest et al., 2017), and pH and *p*CO_2_ could vary by up to 0.7 units and 900 μatm, respectively, over a diel cycle (Santos et al., 2011; Shaw et al., 2012; Chen et al., 2015; Silverman et al., 2015). This environmental variability may greatly confound our current understanding and predictions of OA consequences on marine organisms, especially for those inhabiting high-variance ecosystems (Rivest et al., 2017). Therefore, focus is now shifting to how natural *p*CO_2_ fluctuations will interact with increasing *p*CO_2_ levels to affect the future performance of shallow marine organisms.

Recent evidence suggests that reef calcifiers respond very differently to constant elevated *p*CO_2_ than to oscillating *p*CO_2_ that simulates the daily variations in carbonate chemistry on shallow reefs. For instance, recruits of the reef coral *Seriatopora caliendrum* exposed to ecologically relevant *p*CO_2_ fluctuations exhibited higher rates of calcification and survival compared to those under ambient or high *p*CO_2_ (Dufault et al., 2012). Similarly, diel *p*CO_2_ oscillations totally negate the inhibition of calcification by OA in corals *Acropora formosa* (Chan and Eggins, 2017) and *A. hyacinthus* (Comeau et al., 2014b), and variable *p*CO_2_ partially offset the OA-induced depression in calcification by crustose coralline algae (CCA) *Porolithon onkodes* (Johnson et al., 2014). In contrast, periods of high pH in the daytime and low pH at night act additively with OA to reduce the skeletal growth of adult and juvenile coralline algae *Arthrocardia corymbosa* (Cornwall et al., 2013; Roleda et al., 2015). Another recent study demonstrated that calcification rates of the coral *Goniopora* sp. and the CCA *Hydrolithon reinboldii* exhibited limited response to both OA and extreme pH fluctuations, possibly due to strong control over carbonate chemistry within the calcifying fluid (Cornwall et al., 2018). Differential calcification responses to diurnal *p*CO_2_ fluctuations that are typical on shallow tropical reefs, together with OA, give rise to a clear need for more thorough studies of the influence of dynamic *p*CO_2_ on calcification, especially under acidified seawater.

Furthermore, little is known about the biochemical mechanisms of these observed calcification responses of reef corals to future OA conditions. Symbiotic scleractinian corals are known to calcify faster in the light than in the dark, a phenomenon called “light enhanced calcification” (LEC) (Allemand et al., 2011). Photosynthesis by endosymbionts (i.e., zooxanthellae), which contributes greatly to the energy needs of coral holobiont (Muscatine et al., 1981), has been considered the primary cause of LEC. During daytime, photosynthesis consumes CO_2_ and helps to maintain intracellular pH and elevated ${\mathrm{CO}}_{3}^{2-}$ and aragonite saturation state (Ω_Arg_) (Gibbin et al., 2014). Together, this creates conditions favorable for calcification (McCulloch et al., 2012; DeCarlo et al., 2018). Meanwhile, release of hydroxide ions (OH^-^) from photosynthesis makes the coelenteron an alkaline environment, supporting the titration of the protons (H^+^) produced by calcification (Moya et al., 2008; Comeau et al., 2013a).

Carbonic anhydrase (CA), which catalyzes the inter-conversion between bicarbonate (${\mathrm{HCO}}_{3}^{-}$) and CO_2_, is central to carbon supply for calcification, conversion of metabolic CO_2_ to prevent night acidosis, and carbon concentrating for photosynthesis by zooxanthellae (Leggat et al., 1999, 2002; Moya et al., 2008; Bertucci et al., 2013). Moreover, Ca-ATPase and Mg-ATPase are hypothesized to transport Ca^2+^ and Mg^2+^ into the extracellular calcifying fluid (ECF) and simultaneously remove H^+^ from ECF in reef corals (Ip and Lim, 1991; Ip et al., 1991; Meibom et al., 2004; Zoccola et al., 2004; Allison et al., 2011), thus driving the calcification reaction toward CaCO_3_ precipitation (Al-Horani et al., 2003; Allemand et al., 2011). Although studies have shown that OA could dramatically change the gene expression of CA, Ca-ATPase, and Mg-ATPase (Kaniewska et al., 2012; Vidal-Dupiol et al., 2013; Kurihara et al., 2018), the effects of elevated and fluctuating *p*CO_2_ on the functions of these crucial molecules, and more importantly, the way in which they may coordinate to regulate photosynthesis and calcification under future OA remain largely unexplored.

Further, OA has been demonstrated to increase reactive oxygen species (ROS) generation and induce oxidative stress in calcifying organisms (Matozzo et al., 2013; Mangan et al., 2017; Luz et al., 2018). As the energetic costs of calcification and acid-base regulation are expected to increase as *p*CO_2_ rises (Cohen and Holcomb, 2009), this may reduce the energy availability for ROS scavenging by the antioxidant system, also a high energy-demanding process. Again, current literature provides little information on the effects of seawater acidification on the oxidative stress and antioxidant functioning for scleractinian corals.

To address these critical knowledge gaps, the present study investigated the physiological and biochemical responses of the reef coral *Pocillopora damicornis* to diel *p*CO_2_ fluctuations that are characteristic of their natural settings. *P. damicornis* is a widely distributed and major reef-building coral on reef flats in the Indo-Pacific region and broods symbiotic planula larvae with maternally derived zooxanthellae (Veron, 1993). Several prior studies demonstrated that *P. damicornis* is resistant to OA, with unaffected calcification under high *p*CO_2_ (Comeau et al., 2013b, 2014a, 2015). In our study site Luhuitou fringing reef, calcification by adult *P. damicornis* even responded positively to elevated *p*CO_2_, suggesting the local acclimatization/adaptation to OA (Huang et al., 2014), while the post-settlement calcification and growth are highly susceptible to OA conditions (Jiang et al., 2015, 2018). Here, we extended our use of new recruits of *P. damicornis* and examined the effects of stable and fluctuating OA on a suite of physiological traits, including photochemical performance, survivorship and early development. In addition, oxidative stress and activities of CA, Ca-ATPase and Mg-ATPase putatively involved in photosynthesis and calcification were measured to illuminate the physiological changes of juvenile corals.

## Materials and Methods

### Study Site and *in situ p*CO_2_ Profiling

To quantify the pattern of diel *p*CO_2_ oscillations currently experienced by corals, seawater was continuously pumped from 2 m depth on Luhuitou fringing reef into a flow-through tank on the beach. Seawater *p*CO_2_ was measured *in situ* in the tank using a Picarro CRDS (Cavity Ring-Down Spectroscopy) analyzer. The accuracy of CRDS analyzer was verified by measuring certified reference gas standards, as per the manufacturer’s instructions. Seawater *p*CO_2_ was monitored for 7 days from 20 to 27 August 2017.

### Coral Sampling and Larval Settlement

Ten mature colonies of *P. damicornis* were collected at 2 m depth on Luhuitou reef (N18°12.7′, E109°28.5′) by snorkeling on August 26, 2017. Colonies were transported to the Tropical Marine Biological Research Station, placed into individual 20 L tanks with flow-through seawater at ambient temperature (28.6 ± 0.2°C) and exposed to partially shaded sunlight (noon irradiance, *ca.* 300 μmol photons m^-2^ s^-1^). The outflow of each tank was passed through a cup fitted with a 180 μm mesh on the bottom to trap larvae. Larvae were collected at 08:00 on August 30, 2017 and then pooled across colonies. Groups of approximately 60 larvae were introduced into plastic petri-dishes, and settlement was induced by small chips of crustose coralline algae *Porolithon onkodes*. Twelve hours later, unsettled larvae and algal chips were discarded. Five dishes with a total of approximately 200 primary polyps were allocated to each experimental tank. The newly settled corals were reared for 7 days under three *p*CO_2_ treatments as described below.

### Experimental Setup

Juvenile corals were exposed to three *p*CO_2_ treatments: (1) steady and ambient *p*CO_2_ (Control); (2) steady and elevated *p*CO_2_ (Stable OA); and (3) diurnally fluctuating and elevated *p*CO_2_ (Fluctuating OA). It is important to point out that the ambient *p*CO_2_ of seawater measured here was much higher than the open ocean level. However, it just reflected the current conditions in this costal reef ecosystem and was comparable to the values previously reported at this location (Zhang et al., 2013; Chen et al., 2015; Yan et al., 2016). The *p*CO_2_ of the stable OA treatment was chosen based on the projections by the end of this century under RCP8.5 (Gattuso et al., 2015). The fluctuating OA treatment was created by superimposing the current diurnal variance onto the *p*CO_2_ level predicted for 2100 (Cornwall et al., 2013; Camp et al., 2016). Limited facility precluded an additional, fluctuating treatment at ambient *p*CO_2_, but this limitation did not affect the main objective of this study, i.e., comparing the physiological responses of juvenile corals to stable and oscillatory OA conditions. The experimental *p*CO_2_ treatments were constructed in nine 25-L tanks, with three replicate tanks for each treatment. Each tank was filled with 0.5 μm-filtered and UV-sterilized seawater. Seawater within each tank was well mixed using submerged pumps (350 L h^-1^) and partially (30%) changed at 20:00 every day. Each tank was covered with a transparent lid to minimize gas exchange and maintain experimental *p*CO_2_ levels. The two steady regimes were established by bubbling with ambient air or elevated *p*CO_2_ (1000 ppm), which was achieved by mixing air with CO_2_ using a CO_2_ enricher (CE100B, Ruihua, China). The fluctuating treatment was established by changing *p*CO_2_ settings of the CO_2_ enricher every 6 h. Preliminary high-resolution pH monitoring (every hour for 1 day) showed that this method successfully achieved step-wise and gradual decrease and increase in seawater pH, following a natural daily cycle. Mean pH of the fluctuating OA treatment was comparable to that in the stable OA treatment (7.82 vs. 7.81).

The seawater temperature in each tank was controlled independently with digital temperature controllers and titanium heaters at a targeted value of 29 ± 0.4°C (mean ± SD), which corresponded to the ambient and long-term mean summer temperature at the study site. Light was provided on a 12:12 h light-dark cycle between 07:00 and 19:00 h using T5 fluorescent lamps. The photosynthetically active radiation was about 200 μmol photons m^-2^ s^-1^, approximating that recorded in crevices preferred by juvenile corals at 2–3 m depths on Luhuitou reef (Lei Jiang, unpublished data). Seawater samples (100 mL) were collected from each tank at 06:00 and 18:00 every other day for the measurement of pH, salinity and total alkalinity. pH and salinity were measured with a Thermo Orion 5-star meter and the pH electrode was two-point calibrated with NBS buffers every other day. Total alkalinity (TA) was measured with an automatic titrator (AS-ALK2, Apollo, United States). The carbonate chemistry parameters were calculated using the CO2SYS program and seawater conditions for each treatment are presented in Table 1.

**Table 1:** Mean (±SD) physical and chemical parameters for each treatment.

Treatment	pH_NBS_	Salinity (psu)	TA (μmol kg^-1^)	DIC (μmol kg^-1^)	*p*CO_2_ (μatm)	Ω_Arag_
Control	8.11 ± 0.02	33.4 ± 0.6	2245 ± 87	1981 ± 78	508 ± 32	3.13 ± 0.2
Stable OA	7.81 ± 0.02	33.2 ± 0.4	2195 ± 81	2074 ± 78	1115 ± 77	1.70 ± 0.12
Fluct OA	7.82 ± 0.12	33.3 ± 0.6	2158 ± 126	2032 ± 146	1217 ± 621	1.77 ± 0.73


### Chlorophyll Fluorescence Measurement

The photo-physiology of the symbionts was assessed using four distinct parameters associated with chlorophyll fluorescence. Five recruits from each replicate tank were randomly selected on the last day of the experiment, and a Diving-PAM (Pulse Amplitude Modulated) fluorometer (Walz GmbH, Germany) was used to assess the chlorophyll fluorescence parameters of *in hopite* symbionts. The fiber-optic probe was equipped with a plastic tube to ensure consistent probe orientation and distance of 2 mm between probe and corals. Maximum and effective quantum yields (*F*_v_/*F*_m_ and Δ*F*/*F*_m_′) were measured for the same batch of recruits from each tank using the equations of Genty et al. (1989): *F*_v_/*F*_m_ = (*F*_m_ - *F*_0_)/*F*_m_; Δ*F*/*F*_m_′ = (*F*_m_′ -*F*_t_′)/*F*_m_′, where *F*_m_ = maximum fluorescence yield, *F*_0_ = fluorescence yield in darkness, *F*_t_′ = fluorescence yield in actinic light and *F*_m_′ = maximum fluorescence yield in actinic light. *F*_v_/*F*_m_ was measured at 06:00 to ensure enough time for dark adaptation and relaxation of photochemical quenching. *F*_v_/*F*_m_ provides a measure of the maximum photochemical efficiency of photosystem II (PSII), with substantial declines indicating damage to the photosynthetic apparatus (Jones et al., 1998). Δ*F*/*F*_m_′ was measured at 17:00 in a light-adapted state. This ratio assesses the actual light use efficiency to drive photochemical processes (Maxwell and Johnson, 2000). The depression of Δ*F*/*F*_m_′ relative to *F*_v_/*F*_m_ reflects the extent of non-photochemical quenching (*NPQ*), which is determined as (*F*_m_ -*F*_m_′)/*F*_m_′. Finally, maximum citation pressure over PSII (*Q*_m_) is determined from 1 - [(Δ*F*/*F*_m_′ at 17:00)/(*F*_v_/*F*_m_)], with values close to 0 indicating that most of the reaction centers are open, while values close to 1 denoting mostly closed reaction centers and photo-inhibition (Iglesias-Prieto et al., 2004).

### Survival and Growth

Recruits were checked daily and the number of dead corals was recorded based on loss of polyp tissue and presence of bare skeleton. On the last day of the experiment, 15-20 recruits were randomly selected from each tank, photographed under a dissecting microscope for the growth measurements, and the number of new buds counted for each recruit. Images with a scale bar were analyzed for lateral growth using ImageJ software (National Institutes of Health). Growth was estimated as the rates of change in planar area and number of new polyps over time (Dufault et al., 2012; Jiang et al., 2018). Upon completion of the experiment, 10 recruits were sampled from each tank and analyzed for skeletal weight and ash-free tissue biomass using a Mettler-Toledo ultra-microbalance at an accuracy of ±1 μg according to Anlauf et al. (2011).

### Enzymatic and Oxidative Stress Assays

On the last day of the experiment, 4 groups of 15 recruits from each tank were randomly sampled using sterilized razor blades at 06:00 and 18:00. Two samples from each time were assayed for activities of Ca-ATPase and Mg-ATPase following modified protocols of Chan et al. (1986) and Prazeres et al. (2015). The remaining two samples were used to measure CA activities using the pH drift method (Weis et al., 1989). All samples were immediately snap-frozen in liquid nitrogen, transported on dry-ice to the lab in Guangzhou and preserved at -80°C. Detailed procedures of these assays are provided in the electronic Supplementary Material.

In addition, two batches of 10 recruits were sampled from each tank to analyze the signs of oxidative stress, including catalase (CAT) activity and malondialdehyde (MDA) content. Up-regulation of CAT reflects an organism’s capacity to detoxify ROS, and MDA is an indicator of cellular oxidative damage and lipid peroxidation. CAT was assayed using the Catalase assay kit (Beyotime, China) as per the manufacturer’s instructions. MDA content was determined as the thiobarbituric acid reactive metabolites (Camejo et al., 1998) using a Lipid Peroxidation Assay Kit (Sigma-Aldrich, United States).

### Data Analyses

All response data were initially tested using a nested ANOVA with tank as a random factor nested within *p*CO_2_ treatment. However, as the tank factor was non-significant (electronic Supplementary Material), it was dropped from the statistical model to enhance the power of the analysis (Quinn and Keough, 2002), and the analyses were repeated using corals as independent replicates. To assess the effects of *p*CO_2_ treatments on photo-physiology, growth, CAT activity and lipid peroxidation, one-way ANOVAs were applied with Fisher’s Least Significant Difference (LSD) as planned *post hoc* multiple comparisons. The enzymatic activities were analyzed with two-way ANOVAs followed by Fisher’s LSD, with *p*CO_2_ and time as fixed effects. Further *post hoc* tests for pairwise comparison of the effect of time on the enzymatic activities at each *p*CO_2_ treatment were performed using Student’s *t*-test.

## Results

### Environmental and Experimental Seawater *p*CO_2_

**FIGURE 1:**
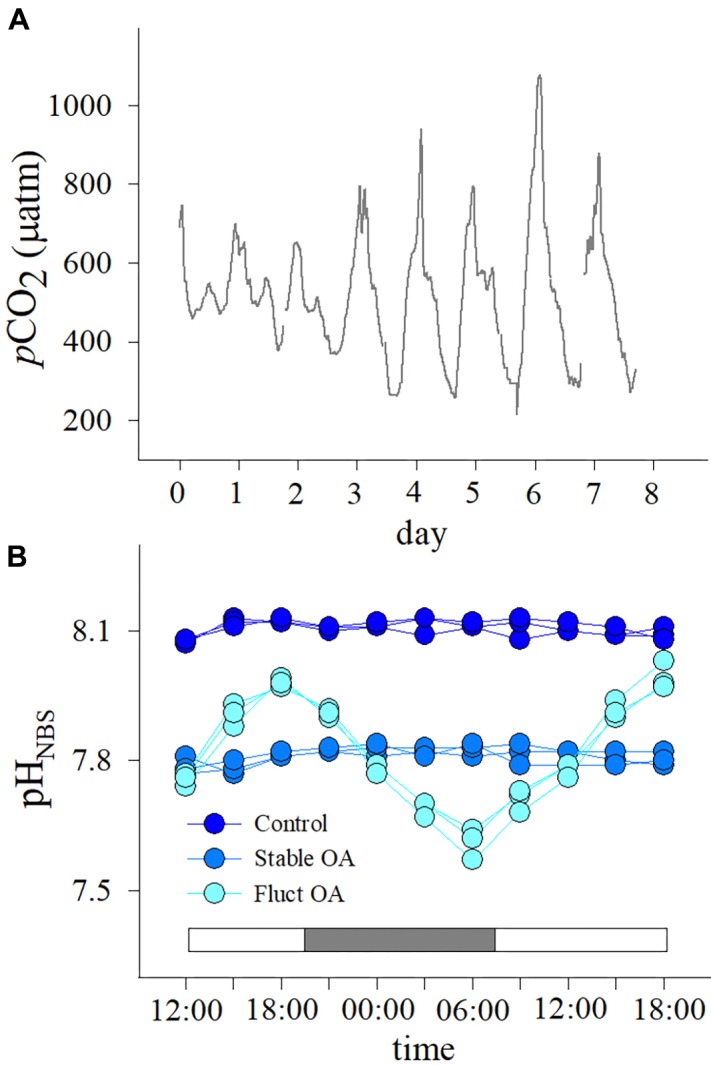
**(A)** Seawater *p*CO_2_ profiles over 7 days at 2 m depth on Luhuitou fringing reef and **(B)** patterns of diurnal variation in seawater pH in the three *p*CO_2_ treatments. The two intervals in **(A)** denote that water sampling and *p*CO_2_ measurement were not possible due to pump failure. White and gray bars in **(B)** indicate light and dark periods, respectively.

Seawater *p*CO_2_ at 2 m depth on Luhuitou fringing reef ranged from 215 to 1077 μatm over 7 days in summer (Figure 1A), averaging 528 ± 163 μatm (mean ± SD). A prominent diel cycle was present, with the lowest value before dusk and the highest value near dawn. Mean diurnal range of seawater *p*CO_2_ was 518 ± 220 μatm (mean ± SD, range: 247–862 μatm).

Diurnal variations in seawater pH for each treatment are shown in Figure 1B. In the fluctuating OA treatment, pH decreased after 18:00 until reaching a minimum at 06:00 the next morning (Figure 1B). The *p*CO_2_ values for the control, stable OA and fluctuating OA treatments were 508 ± 32, 1115 ± 77, and 1217 ± 621 μatm, respectively (mean ± SD, Table 1).

### Survival and Chlorophyll Fluorescence

**FIGURE 2:**
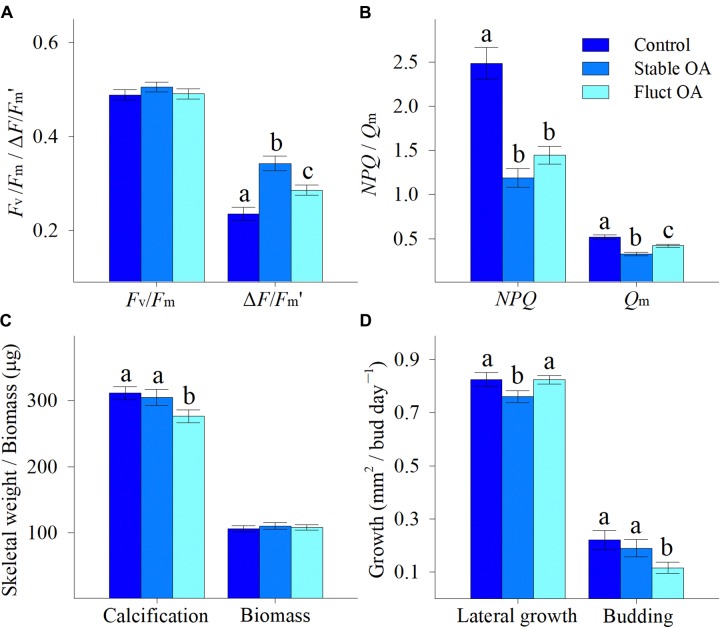
Physiological responses of *Pocillopora damicornis* recruits following 7-day exposure to ambient *p*CO_2_ (Control), steady-high *p*CO_2_ (Stable OA), and fluctuating-high *p*CO_2_ (Fluct OA). **(A)** Maximum and effective quantum yields (*F*_v_/*F*_m_ and Δ*F*/*F*_m_′); **(B)** non-photochemical quenching and maximum citation pressure over PSII (*NPQ* and *Q*_m_); **(C)** calcification and biomass; and **(D)** lateral growth and budding rates. Data are expressed as mean ± SE. Different letters indicate statistically significant differences in mean values among treatments.

Coral survival rate was high and only 2 recruits, one each from the control and stable OA treatments, died during the experiment. There was no significant differences in *F*_v_/*F*_m_ between the *p*CO_2_ treatments, and mean *F*_v_/*F*_m_ ranged from 0.488 to 0.505 (*F*_2,42_ = 0.691, *p* = 0.507; Figure 2A). In contrast, both the stable and fluctuating OA treatments had a significant and positive impact on Δ*F*/*F*_m_′ (*F*_2,42_ = 15.46, *p* < 0.001; Figure 2A). Δ*F*/*F*_m_′ was highest in the stable OA treatment but declined in the fluctuating OA treatment (Figure 2A). Consequently, *NPQ* and *Q*_m_ were significantly reduced at increased *p*CO_2_ (*F*_2,42_ ≥ 21.14, *p* < 0.001), with apparent trends for lower values under stable OA relative to fluctuating OA (Figure 2B).

### Post-settlement Growth

Calcification was marginally affected by *p*CO_2_ (*F*_2,87_ = 3.09, *p* = 0.051), and reduced by 10% in the fluctuating OA treatment compared to the other treatments (Figure 2C). Mean tissue biomass per individual ranged from 106 to 111 μg, and was similar among treatments (*F*_2,87_ = 0.20, *p* = 0.818; Figure 2C). Lateral growth was affected by *p*CO_2_ treatments (*F*_2,155_ = 3.12, *p* = 0.047), and reduced by 8% under stable OA relative to control and fluctuating OA (Figure 2D). *p*CO_2_ treatments significantly influenced asexual budding (*F*_2,155_ = 3.33, *p* = 0.038), and the budding rate in the fluctuating OA treatment was only about half that in the other two treatments (Figure 2D).

### CAT and Lipid Peroxidation

**FIGURE 3:**
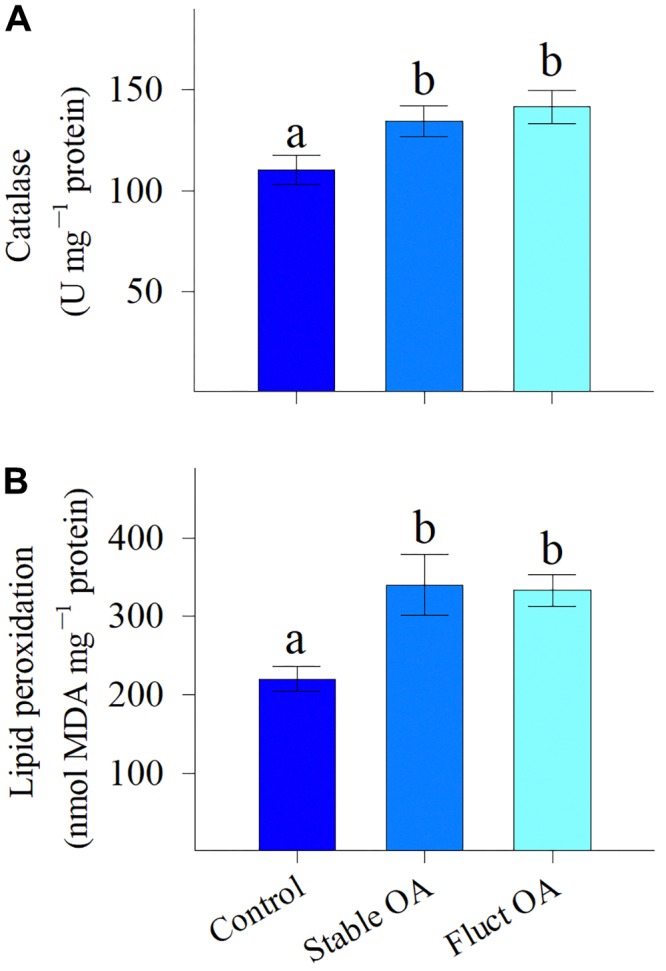
**(A)** Activity of the antioxidant enzyme catalase and **(B)** levels of lipid peroxidation of *P. damicornis* recruits exposed to ambient *p*CO_2_ (Control), steady-high *p*CO_2_ (Stable OA), and fluctuating-high *p*CO_2_ (Fluct OA). Data shown as mean ± SE. Different letters indicate statistically significant differences in mean values among treatments.

Host CAT activity was significantly elevated by OA treatments (*F*_2,15_ = 4.44, *P* = 0.03), but was similar between the stable and fluctuating OA treatments (Figure 3A). There was also a significant effect of *p*CO_2_ treatments on MDA content in host tissue (*F*_2,15_ = 6.39, *P* = 0.01). MDA concentrations were 55 and 51% higher for corals under stable and fluctuating OA than that in the control, respectively (Figure 3B).

### Carbonic Anhydrase

**FIGURE 4:**
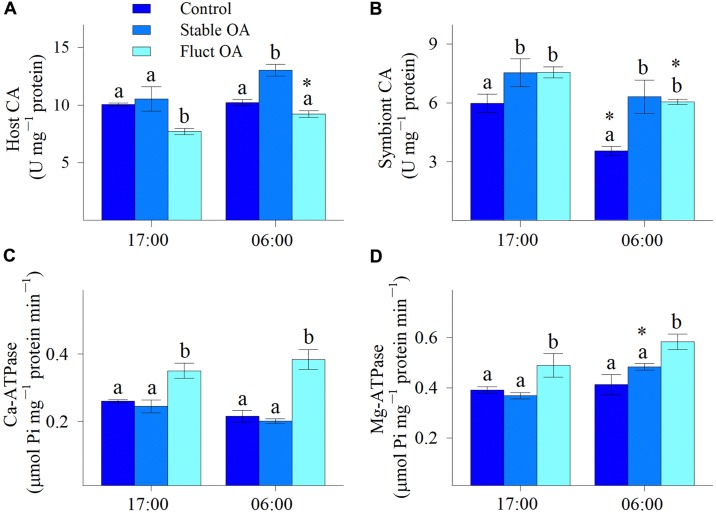
Enzymatic activities of *P. damicornis* recruits exposed to ambient *p*CO_2_ (Control), steady-high *p*CO_2_ (Stable OA), and fluctuating-high *p*CO_2_ (Fluct OA). **(A)** Host CA; **(B)** symbiont CA; **(C)** Ca-ATPase; and **(D)** Mg-ATPase. Data shown as mean ± SE. Different letters indicate statistically significant differences in mean values among treatments, while asterisks denote significantly different means between night and day within each treatment.

Both host and symbiont CA varied across *p*CO_2_ treatments and between night and day, and treatment and time had no significant interactive effects (Table 2). Compared to the control, host CA was significantly elevated in the stable OA treatment and reduced in the fluctuating OA treatment (Figure 4A). *Post hoc* analyses showed that host CA in the fluctuating OA treatment differed significantly between light and dark conditions (Student *t*-test, *df* = 10, *t* = 3.905, *p* = 0.003). Furthermore, corals in both OA treatments exhibited significantly higher symbiont CA activities (Figure 4B). *Post hoc* analyses revealed that symbiont CA in the control and fluctuating OA treatments was significantly lower in the dark than that in the light (Student *t*-test, *df* = 10, *t* = -4.606, *p* = 0.003; *t* = -4.885, *p* = 0.001).

**Table 2:** Statistical results of two-way ANOVAs examining the effects of *p*CO_2_ treatments and time on the activities of carbonic anhydrase (CA), Ca-ATPase and Mg-ATPase of *Pocillopora damicornis* recruits.

Variables	Source of variation	SS	*df*	MS	*F*	*P*
Host CA	*p*CO_2_	66.08	2	33.04	20.47	**<0.001**
	Time	17.37	1	17.37	10.76	**0**.**003**
	*p*CO_ 2_^∗^ Time	8.167	2	4.083	2.530	0.097
	Error	48.42	30	1.614		
Symbiont CA	*p*CO_ 2_	35.43	2	17.72	11.00	**<0.001**
	Time	26.64	1	26.64	16.54	**<0.001**
	*p*CO_2_^∗^ Time	2.380	2	1.190	0.739	0.486
	Error	48.30	30	1.610		
Ca-ATPase	*p*CO_ 2_	0.150	2	0.075	24.00	**<0.001**
	Time	0.003	1	0.003	0.976	0.331
	*p*CO_2_^∗^ Time	0.012	2	0.006	1.912	0.165
	Error	0.094	30	0.003		
Mg-ATPase	*p*CO_ 2_	0.123	2	0.053	6.674	**0**.**002**
	Time	0.053	1	0.061	7.685	**0**.**015**
	*p*CO_2_^∗^ Time	0.015	2	0.007	0.923	0.408
	Error	0.240	30	0.008		


*Significant results are highlighted in bold.*

### Ca-ATPase and Mg-ATPase

In general, there was a significant effect of *p*CO_2_ treatments on the activities of Ca-ATPase and Mg-ATPase (Table 2), both of which were greatly elevated in the fluctuating OA treatment compared to the other 2 treatments. Relative to the control and stable OA treatments, Ca-ATPase activity in the fluctuating OA treatment increased by 35-43% and 78-91% during light and dark periods, respectively (Figure 4C). However, such pattern was less clear for Mg-ATPase activity, which was elevated in the fluctuating OA treatment by 21-41% and 25-33% in dark and light conditions, respectively. Furthermore, Ca-ATPase activity was unaffected by time (Table 2); by contrast, Mg-ATPase activities differed significantly between light and dark conditions (Table 2), largely driven by the higher nighttime Mg-ATPase activities under stable OA (Student *t*-test, *df* = 10, *t* = -5.19, *p* < 0.001; Figure 4D).

## Discussion

The present study revealed significant differences in the physiological responses of juvenile *P. damicornis* exposed to constant and fluctuating OA. Results showed that the fluctuating *p*CO_2_ regime depressed the stimulatory effect of OA on photosynthetic activity. Further, corals significantly up-regulated the proton pumps and antioxidant CAT in the fluctuating OA treatment, in which calcification was only slightly reduced. However, asexual budding declined by 50% and oxidative damage still occurred under fluctuating OA. Together, although the photochemical performance was enhanced by the fluctuating OA treatment, it was unable to fully compensate for the increased energy expense for coral recruits. More importantly, in the fluctuating OA treatment, corals appeared to compromise asexual reproduction and ROS detoxification to sustain skeletal growth, indicating potential trade-offs between calcification and other key physiological processes. These findings suggest that diurnal variability in pH/carbonate chemistry is likely to be an overriding factor influencing and determining the early success and recruitment of corals under future OA. Our study also highlights the importance of considering a broader spectrum of physiological traits in order to accurately and fully characterize the overall change in fitness and the possible trade-offs between different physiological functions when addressing corals’ responses to environmental stress.

### Boosted Photo-Physiology and Potential Involvement of Symbiont CA Under OA

Consistent with our previous findings (Jiang et al., 2015, 2018), this study showed that OA did not influence *F*_v_/*F*_m_, indicating that there was no photo-inhibition or damage to the photosynthetic apparatus. Further, both OA treatments greatly improved the photosynthetic efficiency of *in hospite* symbionts, as evidenced by the elevated Δ*F*/*F*_m_′ and decreased *NPQ* and *Q*_m_. These findings suggest that, under elevated *p*CO_2_, more electrons are being transported for carbon fixation and the photochemical process is more competitive for reaction centers than non-photochemical quenching. A similar photo-physiological response was observed in our previous study on *P. damicornis* recruits exposed to increased *p*CO_2_ (Jiang et al., 2015).

Zooxanthellae possess the type II Rubisco with low CO_2_ affinity, and are therefore carbon-limited under current *p*CO_2_ levels (Leggat et al., 2002). However, CO_2_ enrichment effects on photosynthesis are occasionally observed in reef corals. For example, Noonan and Fabricius (2016) showed that increased *p*CO_2_ significantly promoted photosynthetic productivity in reef coral *S. hystrix* from central Great Barrier Reef, but not in *A. millepora*. In contrast, Strahl et al. (2015) reported that net photosynthesis of *A. millepora* and massive *Porites* spp. from volcanic CO_2_ seeps in Papua New Guinea increased considerably with *p*CO_2_, whereas this phenomenon was not observed in *S. hystrix* and *P. damicornis*. Additionally, parabolic responses of photochemical process to *p*CO_2_ have also been documented (Crawley et al., 2010; Castillo et al., 2014). Hence, photo-physiological responses to OA in reef corals may be species-specific and context-dependent. Moreover, different *p*CO_2_ levels and the diverse DIC utilization modes of symbionts (Brading et al., 2011, 2013) may contribute to these variable and contrasting results.

Corals from Luhuitou reef have periodically encountered acute *p*CO_2_ levels comparable to OA projections for the end of this century. This history of exposure to dynamic and natural extremes of carbonate chemistry could facilitate plasticity and acclimatization of coral host and/or their associated symbionts to future OA. The boosted photo-physiology under OA in the present study is likely an adaptive response, and the CO_2_ fertilization effect might be driven by the increased symbiont CA activities, which could accelerate inter-conversion between ${\mathrm{HCO}}_{3}^{-}$ and CO_2_ and thus DIC transport to thylakoid within symbiont cells. Albeit energy-consuming, these processes would ultimately promote the accumulation of CO_2_ around Ribulose bisphosphate carboxylase/oxygenase (Rubisco) and support photosynthesis (Leggat et al., 1999, 2002). Intriguingly, *NPQ* and *Q*_m_ tended to be higher under fluctuating OA than stable OA, suggesting an increase in non-photochemical process and acidosis of the thylakoid lumen. This probably resulted from the increased energy demand of calcification (discussed below), which would sequentially reduce the availability of ATP for H^+^ removal in dark reactions and trigger acidosis within the thylakoid lumen (Kanazawa and Kramer, 2002).

### Oxidative Stress in Response to OA

Despite the positive photochemical response to OA, we found that both OA regimes elicited a significant and similar increase in activity of the antioxidant CAT. Nevertheless, the elevated CAT activity was not effective against the damaging ROS, and lipid peroxidation still increased under OA, as evidenced by the >50% increases in MDA contents, a specific end-product of the oxidative degradation of lipids. This supports previous reports on a range of calcifying marine invertebrates exposed to OA, including the reef coral *P. capitata* (Soriano-Santiago et al., 2013), the hydrocoral *Millepora alcicornis* (Luz et al., 2018) and the mussel *Mytilus edulis* (Mangan et al., 2017).

Reasons for this oxidative stress under OA conditions still remain unclear, particularly for corals under stable OA with considerable energetic benefits from higher photochemical activity. One parsimonious cause is that the increased photosynthetic electron flux under OA could induce the photo-reduction of oxygen, i.e., operation of the Mehler reaction at higher rates, ultimately resulting in the production of damaging hydrogen peroxide and superoxide which would subsequently diffuse into coral cytoplasm (Miyake and Yokota, 2000). ROS accumulates once the scavenging capacity of antioxidant system is exceeded, causing oxidative damage to a range of cell components, such as lipids, nucleic acids and proteins (Asada, 1999). If this is the case, the oxidative stress as measured by lipid peroxidation would have serious repercussions for the holobiont health and its capacity to cope with chronic OA exposure. Further research is clearly needed to pinpoint the exact mechanism behind the oxidative stress caused by OA in reef corals.

### *p*CO_2_ Regime Dictates Coral Calcification and Host CA Function Under OA

Paradoxically, while stable OA reduced lateral growth, it did not affect calcification, suggesting that linear extension and calcification may be decoupled in newly settled corals under OA. The lack of response in early development to static OA contrasts with prior work reporting that steady declines in pH considerably reduced linear growth and calcification of coral recruits (Albright et al., 2010; Jiang et al., 2015, 2018). The most plausible explanation for this discrepancy is the short exposure duration in this study, during which energy reserves might be adequate to sustain calcification. Unexpectedly, calcification was more adversely affected by fluctuating OA than stable OA, which is in strong contrast to prior studies demonstrating either no, partial or complete mitigation of negative OA effects on calcification in adult corals by diel *p*CO_2_ oscillations (Comeau et al., 2014b; Chan and Eggins, 2017; Cornwall et al., 2018). Furthermore, Dufault et al. (2012) reported that calcification by new recruits of *S. caliendrum* from Hobihu, Taiwan responded positively to diurnally fluctuating *p*CO_2_. It should be noted that the seawater *p*CO_2_ range (365–515 μatm) at the study site of Dufault et al. (2012) was narrower than that at our location (215–1077 μatm). Moreover, OA treatments were more extreme in this study and the nighttime *p*CO_2_ of the fluctuating OA treatment in this study was much higher than that of Dufault et al. (2012). Collectively, these large differences between *p*CO_2_ histories, treatment conditions and also life stages likely account for this magnitude of *p*CO_2_ fluctuations influence on coral skeletal growth.

The greater sensitivity of calcification by coral recruits to fluctuating OA than to stable OA can be attributed to at least three non-exclusive reasons. Firstly, the higher photosynthetic activity under stable OA could better promote and fuel calcification, either through direct energy supply for calcification or through the generation of OH^-^ which help neutralize the H^+^ released from ECF, and thus facilitate a higher pH gradient for CaCO_3_ precipitation (Jokiel, 2011; Comeau et al., 2013a; Gibbin et al., 2014). Secondly, in light of the comparable tissue biomass and the decreased lateral growth in the stable OA treatment, area-normalized biomass was higher in stable OA than fluctuating OA. Hence, the thicker tissue layer of recruits under stable OA could create a better separation between ECF and low-Ω_Arg_ ambient seawater and improve corals’ capacity to modulate the internal chemical microenvironment and buffer external acidification (Krief et al., 2010). Finally, it has been proposed that the night-time storage of DIC mediated by host CA could stimulate daytime calcification of corals under diurnally fluctuating *p*CO_2_ (Dufault et al., 2012). However, our data did not support this hypothesis. Instead, host CA under fluctuating OA was unaffected at night but reduced in light conditions, while host CA under stable OA was significantly up-regulated during the night. Although our study did not distinguish between the proportions of host CA utilized in calcification and photosynthesis, the up-regulation of Ca-ATPase under fluctuating OA (discussed below) most likely points to an increase in host CA functioning in calcification, particularly given that DIC assimilation and Ca^2+^ pumping are tightly coupled in calcification (Tanbutté et al., 1996; Furla et al., 2000; Marshall and Clode, 2003). In other words, the declined host CA activity under fluctuating OA could simply reflect the reduced CA function in DIC uptake for photosynthesis.

This presumption is further evidenced by the increased H^+^ pumping activities under fluctuating OA (discussed below) which would produce more CO_2_ that can be recycled for photosynthesis (Furla et al., 2000; Moya et al., 2008). In this case, DIC uptake by host CA from the external seawater for photosynthesis may be reduced to save energy. On the other hand, the increased host CA in the stable OA treatment at night suggests that more DIC could be transported into holobiont (Moya et al., 2008; Bertucci et al., 2013), and this situation would alleviate the daytime DIC competition between calcification and photosynthesis (Furla et al., 2000), further corroborating the unaltered calcification and higher photochemical efficiency under stable OA. It can be therefore concluded that CA activities within coral holobiont are potentially mediated by both environmental *p*CO_2_ and associated changes in other cellular functions.

### Proton Pumping and the Trade-Off Between Calcification and Polyp Budding

Interestingly, Ca- and Mg-ATPase activities (i.e., H^+^, Ca^2+^, and Mg^2+^ pumping) were unaffected in the stable OA treatment, in which calcification was maintained. Also, by using geochemical proxies to assess the chemical profiles within ECF in response to OA, Cornwall et al. (2018) found no change in Ca^2+^ pumping and calcification rates for the coral *Goniopora* sp. and coralline algae *H. reinboldii* under seawater acidification. To a large extent, the unresponsiveness of Ca- and Mg-ATPases to stable OA here could be ascribed to the enhanced photosynthetic performance, which will produce more OH^-^ for the titration of H^+^ from ECF and thus make H^+^ export easier (Jokiel, 2011; Comeau et al., 2013a; Yuan et al., 2018).

Studies of the role of Ca- and Mg-ATPases in corals’ response to OA have yielded inconsistent results. For instance, Vidal-Dupiol et al. (2013) found that the gene coding for Ca-ATPase was upregulated at pH 7.8 and 7.4 but was down-regulated at pH 7.2 in the coral *P. damicornis*, while Kurihara et al. (2018) reported that gene expression of Ca-ATPase in *A. digitifera* did not change in response to high *p*CO_2_. Direct comparisons between these findings and our results are challenging because gene expression patterns may not reflect the exact content and function of proteins, due to post-translational modifications. On the other hand, de Barros Marangoni et al. (2017) observed a 1.6-fold increase in Ca-ATPase activity in the hydrocoral *M. alcicornis* exposed to acidified seawater (pH < 7.5) for 30 days but not for 16 days; likewise, Prazeres et al. (2015) found that Ca- and Mg-ATPase activities of benthic foraminifera *Amphistegina lessonii* significantly increased (+50%) after 30 days at pH 7.6, while the 15-days exposure exerted no effect. However, another resistant species *Marginopora vertebralis* exhibited unaltered Ca-ATPase activity and a 30% inhibition of Mg-ATPase activity following 30-days exposure to lowered pH (Prazeres et al., 2015). Taken together, treatment duration, together with species-specificity, appears to largely influence the response of these crucial enzymes to OA.

Notably, fluctuating OA elicited significant increases in activities of Ca- and Mg-ATPases, and this means that H^+^ pumping was highly activated to maintain pH within the ECF (pH_CF_). As suggested by Comeau et al. (2018), seawater pH and DIC independently control the pH_CF_ of multiple calcifying species, including *P. damicornis*, and pH_CF_ declined with both decreasing seawater pH and increasing seawater DIC. Therefore, when seawater pH was lowest and [DIC] was highest at night in the fluctuating OA treatment, Ca- and Mg-ATPase activities were up-regulated to elevate pH_CF_ and Ω_Arg_, that is, to create a favorable physiochemical microenvironment for calcification (McCulloch et al., 2012; Cai et al., 2016). Nevertheless, given the up-regulation of H^+^ pumps under fluctuating OA, more metabolic CO_2_ will be generated by mitochondria, further exacerbating the nighttime acidosis of calicoblastic cells and thus making H^+^ removal more difficult and skeleton more prone to dissolution (Furla et al., 2000; Moya et al., 2008; Jokiel, 2011). Surprisingly, activities of Ca- and Mg-ATPases in the fluctuating OA treatment also greatly increased during the daytime when seawater was [H^+^] lowest. The daytime up-regulation of H^+^ pumping under fluctuating OA may simply act as a strategy to promote light calcification and offset the possible night dissolution, thus maintaining overall calcification performance.

Additionally, the enhanced Ca- and Mg-ATPase activities indicate that more Ca^2+^ and Mg^2+^ would be delivered into ECF. It has been recently demonstrated that the ability to increase ECF [Ca^2+^] is a key mechanism enabling OA resistance in the reef coral *P. damicornis* (DeCarlo et al., 2018). Furthermore, Mg^2+^ is an important and basic component of center of calcification which controls the nucleation and serves as the basis for the growth of fibrous aragonite crystals (Meibom et al., 2004; Shapiro et al., 2018). The active [Mg^2+^] elevation would be, therefore, advantageous for calcification under fluctuating OA. However, the stronger biological control of pH, [Ca^2+^] and [Mg^2+^] within ECF was still paralleled by a 10% decline in calcification, suggesting that the negative effects of fluctuating OA on skeletal growth could not be fully counteracted by active H^+^ pumping. Moreover, this appeared to come at a cost on asexual reproduction, with a 50% decline in polyp budding, which is extremely energetically expensive for a newly settled coral (Graham et al., 2013). Thus, the increased cost associated with maintaining calcification under fluctuating OA may severely compromise energy investment in asexual budding. These results, together with our previous findings (Jiang et al., 2015, 2018), reaffirm the notion that energy is preferentially allocated to skeletal growth over asexual reproduction in juvenile corals when subjected to OA. Therefore, the measurement of calcification alone may be inadequate to detect the physiological maladaptation and overall changes in fitness of coral recruits under stressful conditions.

## Conclusion

Overall, this study demonstrated significant impacts of *p*CO_2_ fluctuations on the physiological and biochemical properties of juvenile *P. damicornis* in response to OA, and forged the links between coral physiology and the functions of CA, Ca-ATPase, and Mg-ATPase under different *p*CO_2_ regimes. Crucially, the boosted photosynthetic activity under fluctuating OA was insufficient to satisfy the increased energy demand for calcification, potentially causing disproportional energy allocation and arresting other key physiological processes, such as asexual budding and antioxidant system. Evidently, fluctuating OA is more energetically costly than stable OA for the maintenance of newly settled corals. Since diel *p*CO_2_ oscillations are expected to become more pronounced (Shaw et al., 2013), future OA conditions are likely to be more detrimental to the post-settlement development and early success of corals than predicted, especially in highly dynamic costal reefs.

## Data Availability Statement

All datasets associated with this study are included in the manuscript and the Supplementary Files.

## Author Contributions

LJ and HH conceived and designed the study. LJ conducted the experiments and performed the laboratory analyses. LJ analyzed the data and drafted the manuscript. Y-JG, FZ, Y-YZ, LM, X-CY, X-ML, G-WZ, M-LG, LC, J-SL, P-YQ, and HH contributed to lab analysis and interpretation of the results. All authors commented on the draft and gave final consent for publication.

## Conflict of Interest Statement

The authors declare that the research was conducted in the absence of any commercial or financial relationships that could be construed as a potential conflict of interest.
