# Supplementary material for: Diurnally Fluctuating pCO2 Modifies the Physiological Responses of Coral Recruits Under Ocean Acidification
Source: Front Physiol. 2019 Jan 11;9:1952. doi: 10.3389/fphys.2018.01952 (PMC6340097; doi:10.3389/fphys.2018.01952)
Supplement: Supplementary file 1 [file Data_Sheet_1.pdf]

Additional methods and dataset associated with Jiang et al. Diurnally fluctuating *p*CO_2 modifies the physiological responses of coral recruits under ocean acidification.

Detailed procedures of enzymatic assays.

(1) Carbonic anhydrase assay

Carbonic anhydrase (CA) assays were performed following Weis *et al.* [1] with some modifications. Briefly, samples were thawed on ice and homogenized in 1 ml filtered and sterilized seawater (FSW) using a hand-held motor and sterilized pestles which have been chilled in liquid nitrogen. The slurry was centrifuged at 12600 g (4 ℃) for 10min, and the supernatant was decanted to a new tube. The pellet was re-suspended in FSW and left for 1 min to allow the precipitation of skeletal material. The upper phase containing symbiont cells was transferred to a new tube, and centrifuged and washed for 2 times as described previously, after which it was re-suspended in 1 ml FSW and sonicated for 10 min on ice to break the symbiont cells. Microscopic observations confirmed that over 95% of the cells were broken. For each fraction, an aliquot of 400 μl homogenate was transferred to a new glass vial and diluted 1:1 with cold Tris-HCl buffer (pH 8.2, containing 50mM Tris, 5 mM EDTA, 5 mM dithiothreitol (DDT) and 10 mM $MgSO_4$). An additional 1 ml cold buffer was further added. CA activity was measured by the decrease of pH resulting from the hydration of CO_2 to HCO_3^- and H^+. The test sample was equipped with a dual temperature- pH electrode (Mettler-Toledo) and left to equilibrate to 4 ℃ on ice using a magnetic stirrer. When pH stabilizing and temperature reaching 4 ℃, 1 ml of CO_2-saturated distilled water was added to each sample and the reduction of pH within 1 minute was recorded. To control for pH change not associated with CA activity, another 0.4 ml of each fraction was boiled for 10 min to denature the enzyme. As a negative control, the assay was performed with boiled homogenate in a same manner as aforementioned. CA activity was calculated as the Δ pH of the animal or algal homogenate minus the Δ pH of the boiled homogenate and normalized to protein content. Protein concentration was determined via Braford assay [2] using a commercial reagent kit (Sigma-Aldrich,

USA).

(2) Ca-ATPase and Mg-ATPase

Samples containing 15 corals were thawed on ice and homogenized in 200 µl Tris buffer (500 mM sucrose, 150 mM KCl, 20 mM Tris, 1 mM dithiothreitol and 0.1 mM phenylmethylsulfonyl, pH 7.6). Homogenates were then centrifuged at 12600g for 15 min at 4 ℃. The supernatant was transferred to a new tube and an aliquot of 50 µl was preserved for protein determination using Braford assay (Sigma-Aldrich, USA). Ca- and Mg-ATPase were measured according to protocols originally developed by Chan *et al.* [3] and Busacker & Chavi [4] and modified by Prazeres *et al.* [5]. The working buffer for Ca-ATPase contained 80 mM NaCl, 20 mM of Tris-Base, 15 mM KCl and 15 mM $CaCl_2$. Mg-ATPase was measured using a similar working buffer, where $CaCl_2$ was replaced by $MgCl_2$ at the same concentration while pH was adjusted to 8.1. Sample homogenate (20 µl) were mixed with 250 µl of working buffer containing 1 mM of ouabain. The reaction was started by the addition of 30 µl ATP stock solution (30 mM), and then the mixture was incubated at 30 ℃ for 30 min. The reaction was stopped directly by the addition of the Malachite Green Reagent in a Phosphate Assay Kit (Sigma-Aldrich, USA). Three technical replicates were set for each sample. The inorganic phosphate (Pi) released by the enzyme activity was determined based on a colorimetric method [6] using a Phosphate Assay Kit (Sigma-Aldrich, USA), and calculated using a standard curve constructed with 1 mM Pi standards. Measurement was taken at 620 nm using a microplate reader (EnSight, PerkinElmer). The homogenization buffer was used as a blank control [5]. Ca/Mg-ATPase activities were normalized to total protein content and expressed as µmoles Pi mg $protein^{-1}$ min^{-1}.

References:

[1] Weis, V.M., Smith, G.J. & Muscatine, L. 1989 A “CO_2 supply” mechanism in zooxanthellate cnidarians: role of carbonic anhydrase. *Marine Biology* **100**, 195-202.

[2] Bradford, M.M. 1976 A rapid and sensitive method for the quantitation of microgram quantities of protein utilizing the principle of protein-dye binding. *Analytical Biochemistry* **72**, 248-254. (doi:https://doi.org/10.1016/0003-2697(76)90527-3).

[3] KM, C., D, D. & KD, J. 1986 A direct colorimetric assay for Ca^{2+}-stimulated ATPase activity. *Analytical Biochemistry* **157**, 375-380.
[4] Busacker, G.P. & Chavin, W. 1981 Characterization of Na^{+}+K^{+}-ATPases and Mg^{2+}-ATPases from the gill and the kidney of the goldfish (*Carassius auratus* L.). *Comparative Biochemistry and Physiology Part B: Comparative Biochemistry* **69**, 249-256. (doi:https://doi.org/10.1016/0305-0491(81)90237-6).
[5] Prazeres, M., Uthicke, S. & Pandolfi, J.M. 2015 Ocean acidification induces biochemical and morphological changes in the calcification process of large benthic foraminifera. *Proceedings of the Royal Society B: Biological Sciences* **282**. (doi:10.1098/rspb.2014.2782).
[6] Fiske, C.H. & Subbarow, Y. 1925 The colorimetric determination of phosphorus. *Journal of Biological Chemistry* **66**, 375-400.

Dataset

Table S1 Photophysiology of *Pocillopora damicornis* recruits under three pCO_2 treatments

Treatment	Tank	Maximum quantum yield (F_v/F_m)	Effective quantum yield ($\Delta F/F_m'$)	Non-photochemical quenching (*NPQ*)	Maximum excitation pressure over PSII (Q_m)
Control	1	0.47	0.17	3.17	0.63
Control	1	0.5	0.2	2.58	0.61
Control	1	0.54	0.34	1.14	0.36
Control	1	0.55	0.21	2.46	0.61
Control	1	0.4	0.24	2.15	0.4
Control	2	0.53	0.29	2.56	0.46
Control	2	0.48	0.18	2.4	0.62
Control	2	0.52	0.27	2.47	0.47
Control	2	0.45	0.23	2.64	0.49
Control	2	0.43	0.14	4.11	0.67
Control	3	0.44	0.23	2.92	0.48
Control	3	0.51	0.25	2.67	0.5
Control	3	0.5	0.19	2.62	0.61
Control	3	0.48	0.26	1.95	0.47
Control	3	0.52	0.31	1.44	0.4
StableOA	1	0.53	0.45	0.73	0.15
StableOA	1	0.49	0.33	1.14	0.34
StableOA	1	0.54	0.37	0.79	0.31
StableOA	1	0.54	0.35	1.82	0.35
StableOA	1	0.46	0.26	2.08	0.44
StableOA	2	0.54	0.39	0.98	0.27
StableOA	2	0.52	0.34	1.43	0.33
StableOA	2	0.43	0.28	1.21	0.36
StableOA	2	0.46	0.3	1.03	0.36
StableOA	2	0.54	0.45	0.65	0.17
StableOA	3	0.52	0.36	0.79	0.31
StableOA	3	0.43	0.24	1.37	0.44
StableOA	3	0.53	0.34	1.48	0.37
StableOA	3	0.54	0.34	1.13	0.36
StableOA	3	0.51	0.34	1.19	0.33
FluctuatingOA	1	0.5	0.32	0.81	0.36
FluctuatingOA	1	0.56	0.36	1.36	0.36
FluctuatingOA	1	0.47	0.22	1.94	0.53
FluctuatingOA	1	0.55	0.33	1.38	0.4
FluctuatingOA	1	0.54	0.3	1.7	0.44
FluctuatingOA	2	0.53	0.32	0.98	0.39

FluctuatingOA	2	0.51	0.24	2.25	0.52
FluctuatingOA	2	0.51	0.28	1.54	0.45
FluctuatingOA	2	0.46	0.28	1.49	0.39
FluctuatingOA	2	0.43	0.26	1.76	0.38
FluctuatingOA	3	0.43	0.24	1.42	0.46
FluctuatingOA	3	0.45	0.25	1.36	0.44
FluctuatingOA	3	0.46	0.24	1.58	0.49
FluctuatingOA	3	0.45	0.3	1.34	0.34
FluctuatingOA	3	0.51	0.33	0.79	0.34

Table S2 Growth of *Pocillopora damicornis* recruits under three pCO_2 treatments

Treatment	Tank	Lateral growth (mm^2 day^{-1})	Budding rate (new polyp day^{-1})	Treatment	Tank	Biomass (μg $recruit^{-1}$)	Calcification (μg $recruit^{-1}$)
Control	1	0.87	0	Control	1	121	367
Control	1	0.79	0.29	Control	1	88	259
Control	1	0.85	0.57	Control	1	82	210
Control	1	1.08	0.57	Control	1	81	307
Control	1	0.62	0.29	Control	1	104	362
Control	1	0.84	0.29	Control	1	122	406
Control	1	1.22	0.29	Control	1	129	295
Control	1	0.75	0	Control	1	106	242
Control	1	0.94	0.14	Control	1	85	262
Control	1	0.74	0	Control	1	97	280
Control	1	0.82	0.43	Control	2	114	320
Control	1	0.98	0	Control	2	80	284
Control	1	0.65	0	Control	2	120	351
Control	1	0.66	0	Control	2	102	313
Control	2	0.76	0	Control	2	65	256
Control	2	0.92	0	Control	2	95	344
Control	2	0.8	0.29	Control	2	101	283
Control	2	0.77	0.29	Control	2	139	370
Control	2	0.93	0.57	Control	2	107	382
Control	2	0.64	0	Control	2	97	292
Control	2	0.71	0.14	Control	2	121	344
Control	2	0.73	0.29	Control	2	153	440
Control	2	1.23	0.43	Control	3	105	335
Control	2	0.84	0	Control	3	93	259
Control	2	0.8	0.29	Control	3	83	346
Control	2	0.74	0	Control	3	97	290
Control	2	0.64	0.29	Control	3	97	324
Control	2	0.81	0.29	Control	3	103	284
Control	3	0.77	0.14	Control	3	100	260

Control	3	0.9	0.71	Control	3	205	278
Control	3	0.62	0.14	StableOA	1	144	335
Control	3	0.64	0.43	StableOA	1	121	326
Control	3	0.87	0.86	StableOA	1	98	315
Control	3	1.19	0	StableOA	1	95	273
Control	3	0.75	0.14	StableOA	1	156	345
Control	3	0.95	0.57	StableOA	1	106	312
Control	3	0.86	0	StableOA	1	129	373
Control	3	0.66	0	StableOA	1	139	363
Control	3	0.73	0	StableOA	1	96	321
Control	3	1.24	0.29	StableOA	2	145	362
Control	3	0.75	0	StableOA	2	107	346
Control	3	1.06	0.71	StableOA	2	83	131
Control	3	0.66	0	StableOA	2	101	291
Control	3	0.51	0	StableOA	2	74	250
StableOA	1	0.66	0	StableOA	2	55	191
StableOA	1	0.86	0	StableOA	2	104	292
StableOA	1	1	0.29	StableOA	2	86	244
StableOA	1	0.92	0.43	StableOA	2	128	370
StableOA	1	0.66	0	StableOA	2	88	190
StableOA	1	0.65	0	StableOA	3	151	378
StableOA	1	0.9	0.57	StableOA	3	131	386
StableOA	1	0.68	0	StableOA	3	140	326
StableOA	1	1.05	0.43	StableOA	3	120	285
StableOA	1	0.6	0.29	StableOA	3	102	303
StableOA	1	0.7	0.57	StableOA	3	128	343
StableOA	1	0.79	0.14	StableOA	3	88	301
StableOA	1	0.9	0.29	StableOA	3	118	356
StableOA	1	0.65	0	StableOA	3	85	291
StableOA	1	0.46	0	StableOA	3	139	381
StableOA	1	0.85	0.71	StableOA	3	58	172
StableOA	1	0.65	0.14	FluctuatingOA	1	112	278
StableOA	1	0.88	0	FluctuatingOA	1	117	227
StableOA	2	0.94	0.43	FluctuatingOA	1	118	269
StableOA	2	0.89	0	FluctuatingOA	1	136	305
StableOA	2	0.85	0.14	FluctuatingOA	1	120	275
StableOA	2	0.66	0.43	FluctuatingOA	1	91	226
StableOA	2	0.46	0	FluctuatingOA	1	98	283
StableOA	2	1.04	0	FluctuatingOA	1	119	351
StableOA	2	1.02	0.29	FluctuatingOA	1	121	293
StableOA	2	0.69	0.14	FluctuatingOA	1	77	253
StableOA	2	1.01	0.57	FluctuatingOA	2	111	308
StableOA	2	0.73	0.14	FluctuatingOA	2	103	284

StableOA	2	0.82	0	FluctuatingOA	2	131	297
StableOA	2	0.78	0	FluctuatingOA	2	101	304
StableOA	2	0.59	0	FluctuatingOA	2	147	333
StableOA	2	0.91	0.71	FluctuatingOA	2	104	306
StableOA	2	0.53	0.29	FluctuatingOA	2	58	223
StableOA	2	0.7	0	FluctuatingOA	2	101	291
StableOA	2	0.9	0	FluctuatingOA	2	127	316
StableOA	2	0.46	0	FluctuatingOA	2	71	295
StableOA	3	0.78	0.14	FluctuatingOA	3	140	276
StableOA	3	0.64	0	FluctuatingOA	3	94	335
StableOA	3	0.79	0	FluctuatingOA	3	125	320
StableOA	3	0.47	0	FluctuatingOA	3	103	301
StableOA	3	0.92	0.86	FluctuatingOA	3	137	337
StableOA	3	0.51	0	FluctuatingOA	3	74	98
StableOA	3	0.72	0.43	FluctuatingOA	3	100	268
StableOA	3	0.8	0	FluctuatingOA	3	124	238
StableOA	3	0.87	0	FluctuatingOA	3	83	135
StableOA	3	0.92	0.14	FluctuatingOA	3	101	273
StableOA	3	0.99	0.71				
StableOA	3	0.41	0.29				
StableOA	3	0.51	0.29				
StableOA	3	0.8	0				
StableOA	3	0.65	0				
StableOA	3	0.72	0				
StableOA	3	0.73	0				
StableOA	3	0.88	0.14				
StableOA	3	0.84	0.43				
FluctuatingOA	1	0.99	0.29				
FluctuatingOA	1	0.7	0				
FluctuatingOA	1	0.87	0				
FluctuatingOA	1	0.89	0.14				
FluctuatingOA	1	0.87	0				
FluctuatingOA	1	0.88	0				
FluctuatingOA	1	1.01	0				
FluctuatingOA	1	0.78	0				
FluctuatingOA	1	0.69	0				
FluctuatingOA	1	0.66	0				
FluctuatingOA	1	0.8	0				
FluctuatingOA	1	1.06	0.29				
FluctuatingOA	1	0.8	0				
FluctuatingOA	1	1.04	0				
FluctuatingOA	1	0.86	0				
FluctuatingOA	1	0.84	0.57				

FluctuatingOA	1	0.81	0.29				
FluctuatingOA	1	1.27	0				
FluctuatingOA	1	0.84	0.29				
FluctuatingOA	1	0.56	0.43				
FluctuatingOA	2	0.71	0.29				
FluctuatingOA	2	0.83	0				
FluctuatingOA	2	0.84	0				
FluctuatingOA	2	0.87	0.14				
FluctuatingOA	2	0.85	0.57				
FluctuatingOA	2	0.81	0.43				
FluctuatingOA	2	0.74	0				
FluctuatingOA	2	0.93	0				
FluctuatingOA	2	0.7	0				
FluctuatingOA	2	0.75	0				
FluctuatingOA	2	0.9	0				
FluctuatingOA	2	0.81	0				
FluctuatingOA	2	0.89	0				
FluctuatingOA	2	0.87	0.14				
FluctuatingOA	2	0.87	0.14				
FluctuatingOA	2	0.82	0				
FluctuatingOA	2	0.63	0				
FluctuatingOA	2	0.67	0				
FluctuatingOA	2	0.73	0.29				
FluctuatingOA	2	0.72	0.29				
FluctuatingOA	3	0.71	0.14				
FluctuatingOA	3	0.82	0				
FluctuatingOA	3	0.88	0				
FluctuatingOA	3	0.76	0				
FluctuatingOA	3	0.75	0				
FluctuatingOA	3	0.88	0				
FluctuatingOA	3	0.87	0.29				
FluctuatingOA	3	0.96	0				
FluctuatingOA	3	0.89	0				
FluctuatingOA	3	0.72	0				
FluctuatingOA	3	0.84	0.14				
FluctuatingOA	3	0.7	0.43				
FluctuatingOA	3	0.8	0.14				
FluctuatingOA	3	0.62	0.43				
FluctuatingOA	3	0.76	0				
FluctuatingOA	3	0.82	0.14				
FluctuatingOA	3	0.93	0.29				
FluctuatingOA	3	0.73	0.14				
FluctuatingOA	3	0.97	0.14				

Table S3 Catalase activities and lipid peroxidation levels of *Pocillopora damicornis* recruits under three pCO_2 treatments

Treatment	Tank	Catalase (U mg^{-1} protein)	Lipid peroxidation (nmol MDA mg^{-1} protein)
Control	1	96.15	223.52
Control	1	121.04	186.68
Control	2	89.87	197.83
Control	2	138.68	241.77
Control	3	108.53	184.43
Control	3	108.18	286.05
StableOA	1	143.62	473.01
StableOA	1	142.28	249.4
StableOA	2	115.08	333.86
StableOA	2	139.28	310.72
StableOA	3	107.41	243.41
StableOA	3	157.05	430.99
FluctuatingOA	1	131.01	424.99
FluctuatingOA	1	153.35	280.06
FluctuatingOA	2	144.63	330.05
FluctuatingOA	2	130.8	313.2
FluctuatingOA	3	115.97	329.71
FluctuatingOA	3	172.32	319.86

Table S4 Activities of carbonic anhydrase (CA), Ca-ATPase and Mg-ATPase of *Pocillopora damicornis* recruits under three pCO_2 treatments

Time	Treatment	Tank	Host CA (U mg^{-1} protein)	Symbiont CA (U mg^{-1} protein)	Ca-ATPase (μmol Pi mg^{-1} protein min^{-1})	Mg-ATPase (μmol Pi mg^{-1} protein min^{-1})
night	Control	1	10.96	4.4	0.26	0.45
night	Control	1	9.23	3.24	0.19	0.31
night	Control	2	10.3	4.13	0.19	0.55
night	Control	2	9.84	3.3	0.18	0.28
night	Control	3	10.2	3.3	0.3	0.32
night	Control	3	10.78	2.87	0.17	0.56
night	StableOA	1	13.44	7.25	0.17	0.45
night	StableOA	1	13	4.16	0.21	0.51
night	StableOA	2	13.93	8.53	0.19	0.54
night	StableOA	2	10.66	3.38	0.22	0.42
night	StableOA	3	12.87	6.54	0.22	0.49
night	StableOA	3	14.24	7.98	0.2	0.49
night	FluctuatingOA	1	8.22	6.56	0.4	0.57

night	FluctuatingOA	1	9.67	5.89	0.33	0.52
night	FluctuatingOA	2	9.38	5.82	0.4	0.47
night	FluctuatingOA	2	8.49	6.07	0.54	0.73
night	FluctuatingOA	3	9.65	6.26	0.32	0.55
night	FluctuatingOA	3	9.85	5.66	0.31	0.65
day	Control	1	10.03	7.15	0.27	0.36
day	Control	1	9.59	6.76	0.24	0.4
day	Control	2	10.13	5.04	0.27	0.37
day	Control	2	9.85	4.19	0.24	0.42
day	Control	3	10.59	6.71	0.26	0.34
day	Control	3	10.1	5.97	0.28	0.45
day	StableOA	1	10.15	9.76	0.26	0.4
day	StableOA	1	8.2	6.36	0.32	0.3
day	StableOA	2	8.02	7.33	0.17	0.38
day	StableOA	2	14.66	6.08	0.21	0.37
day	StableOA	3	12.45	6.03	0.22	0.4
day	StableOA	3	9.7	9.65	0.3	0.35
day	FluctuatingOA	1	6.65	6.49	0.31	0.64
day	FluctuatingOA	1	7.26	7.01	0.38	0.31
day	FluctuatingOA	2	8.37	8.18	0.45	0.54
day	FluctuatingOA	2	8.08	7.55	0.32	0.5
day	FluctuatingOA	3	8.16	7.91	0.26	0.61
day	FluctuatingOA	3	7.72	8.16	0.38	0.32

Statistical results of nested analyses

Table S5. Statistical results of nested ANOVA examining the effects of pCO_2 on the photo-physiology, early development and oxidative stress of *P. damicornis* recruits.

Source of variation	SS	*df*	MS	*F*	*P*
F_v/F_m					
pCO_2	0.0024	2	0.0012	0.69	0.508
Tank (pCO_2)	0.0105	6	0.0018	0.99	0.446
Error	0.0636	36	0.0018		
$\Delta F/F_m'$					
pCO_2	0.086	2	0.043	14.1	0.001
Tank (pCO_2)	0.008	6	0.001	0.40	0.871
Error	0.109	36	0.003		
NPQ					
pCO_2	14.17	4	7.084	25.9	<0.001
Tank (pCO_2)	1.314	6	0.219	0.80	0.575
Error	9.825	36	0.273		
Q_m					
pCO_2	0.278	2	0.139	19.3	<0.001
Tank (pCO_2)	0.017	6	0.003	0.38	0.884
Error	0.263	36	0.007		
Skeletal weight					
pCO_2	18912	2	9456	3.02	0.055
Tank (pCO_2)	37643	6	6274	2.00	0.075
Error	254027	81	3136		
Biomass					
pCO_2	256.6	2	128	0.20	0.818
Tank (pCO_2)	3408	6	568	0.89	0.506
Error	51638	81	637		
Lateral growth					
pCO_2	0.147	2	0.072	3.01	0.052
Tank (pCO_2)	0.073	6	0.012	0.51	0.801
Error	0.01	149	0.024		
Budding					
pCO_2	0.300	2	0.150	3.16	0.045
Tank (pCO_2)	0.037	6	0.006	0.13	0. 929
Error	7.078	149	0.047		
Catalase					
pCO_2	3143	2	1571	2.85	0.110
Tank (pCO_2)	347	6	58	0.11	0.994
Error	4959	9	551		
Lipid peroxidation					
pCO_2	54501	2	27251	4.06	0.055

Source of variation	SS	*df*	MS	*F*	*P*
Tank (pCO_2)	3607	6	601	0.09	0.996
Error	60362	9	6707		

Table S6. Statistical results of nested ANOVA examining the effects of time and pCO_2 on the activities of carbonic anhydrase, Ca-ATPase and Mg-ATPase of *P. damicornis* recruits.

Variables	Source of variation	SS	*df*	MS	*F*	*P*
Host CA	pCO_2	66.08	2	33.04	15.68	<0.001
	Time	17.36	1	17.36	8.24	0.010
	Tank (pCO_2)	4.695	6	0.782	0.37	0.888
	pCO_2 * Time	8.156	2	4.078	1.94	0.173
	Tank (pCO_2) * Time	5.757	6	0.959	0.46	0.832
	Error	37.93	18	2.107		
Symbiont CA	pCO_2	66.08	2	17.75	9.18	0.002
	Time	26.68	1	26.68	13.8	0.002
	Tank (pCO_2)	6.576	6	1.096	0.57	0.751
	pCO_2 * Time	2.374	2	1.187	0.61	0.552
	Tank (pCO_2) * Time	6.895	6	1.149	0.59	0.731
	Error	34.81	18	1.934		
Ca-ATPase	pCO_2	0.149	2	0.074	26.9	<0.001
	Time	0.003	1	0.003	1.16	0.295
	Tank (pCO_2)	0.031	6	0.005	1.90	0.136
	pCO_2 * Time	0.012	2	0.006	2.22	0.137
	Tank (pCO_2) * Time	0.012	6	0.002	0.72	0.639
	Error	0.050	18	0.003		
Mg-ATPase	pCO_2	0.121	2	0.061	4.62	0.024
	Time	0.054	1	0.054	4.16	0.056
	Tank (pCO_2)	0.008	6	0.001	0.11	0.994
	pCO_2 * Time	0.015	2	0.007	0.57	0.577
	Tank (pCO_2) * Time	0.004	6	0.001	0.05	0.999
	Error	0.236	18	0.013		
